# Limited support for a direct connection between prebiotics and intestinal permeability – a systematic review

**DOI:** 10.1007/s10719-024-10165-8

**Published:** 2024-09-17

**Authors:** Binayak Acharya, Marthe Tofthagen, Marissa L. Maciej-Hulme, Michal Rachel Suissa, Niclas G. Karlsson

**Affiliations:** Department of Life Sciences and Health, Faculty of Health Sciences, Oslo Metropolitan University, St. Olavs Plass, P.O. Box 4, N-0130 Oslo, Norway

**Keywords:** Mucus, Prebiotic carbohydrates, Microbiota, Dysbiosis, Leaky gut, Intestinal permeability

## Abstract

The intestinal barrier is a selective interface between the body´s external and the internal environment. Its layer of epithelial cells is joined together by tight junction proteins. In intestinal permeability (IP), the barrier is compromised, leading to increased translocation of luminal contents such as large molecules, toxins and even microorganisms. Numerous diseases including Inflammatory Bowel Disease (IBD), Coeliac disease (CD), autoimmune disorders, and diabetes are believed to be associated with IP. Dietary interventions, such as prebiotics, may improve the intestinal barrier. Prebiotics are non-digestible food compounds, that promote the growth and activity of beneficial bacteria in the gut. This systematic review assesses the connection between prebiotic usage and IP. PubMed and Trip were used to identify relevant studies conducted between 2010–2023. Only six studies were found, which all varied in the characteristics of the population, study design, and types of prebiotics interventions. Only one study showed a statistically significant effect of prebiotics on IP. Alteration of intestinal barrier function was measured by lactulose/mannitol, chromium-labelled Ethylenediaminetetraacetic acid (^51^Cr-EDTA), lactulose/rhamnose, and sucralose/erythritol excretion as well as zonulin and glucagon-like peptide 2 levels. Three studies also conducted gut microbiota assessment, and one of them showed statistically significant improvement of the gut microbiome. This study also reported a decrease in zonulin level. The main conclusion from this review is that there is a lack of human studies in this important field. Futhermore, large population studies and using standardized protocols, would be required to properly assess the impact of prebiotic intervention and improvement on IP.

**Supplementary Information:**

The online version contains supplementary material available at 10.1007/s10719-024-10165-8.

## Background

### Large intestine and intestinal permeability (IP)

**Fig. 1:**
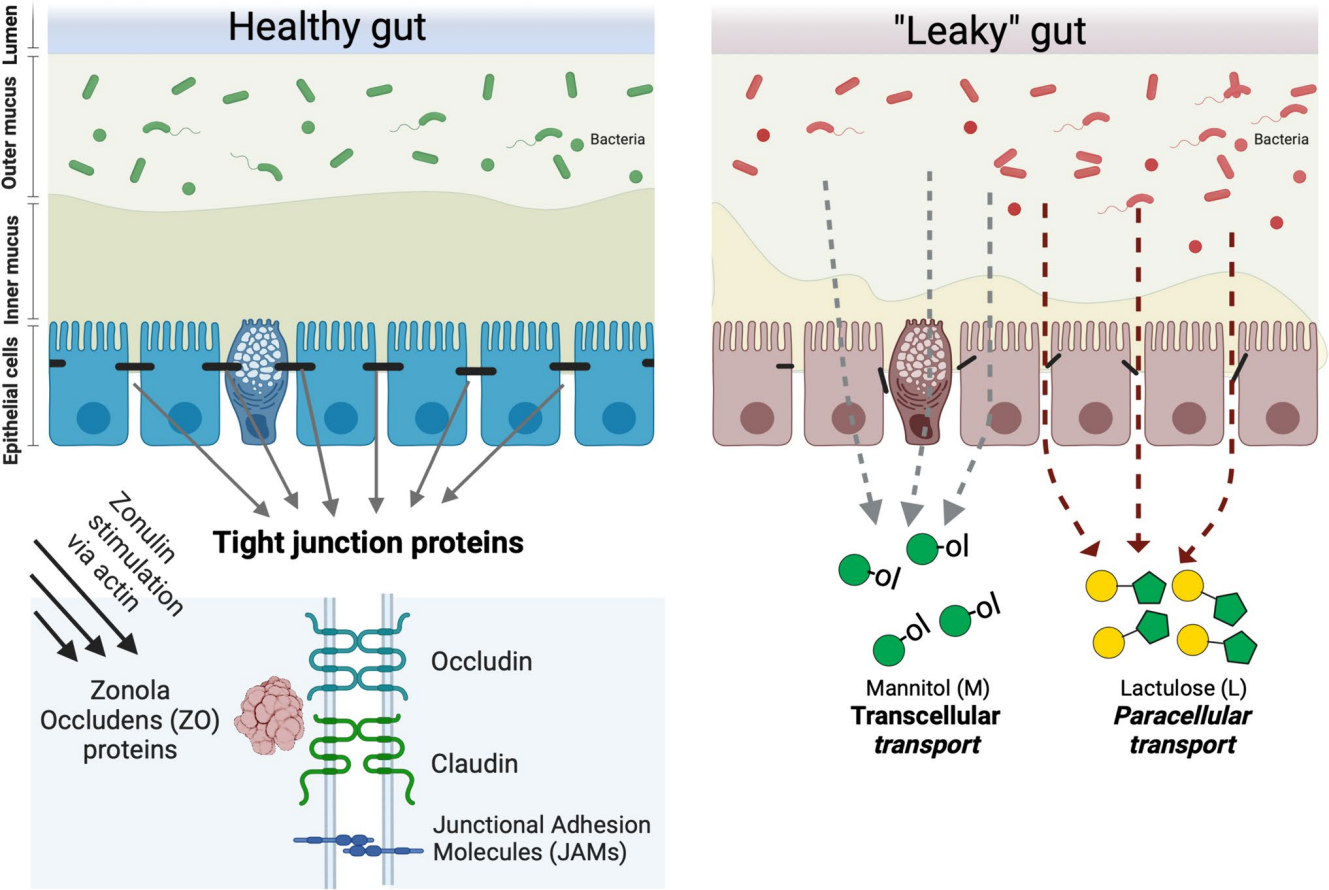
The intestinal barrier. Left side displays a model of a healthy colon, where the turn over of the mucus production is syncronised with the degradation and removal of the mucus barrier. This causes a symbiotic relationship with the microbiota as the dynamic reneveval of the mucus generates an outer mucus layer providing the habitat for the commensal bacteria while an inner mucus layer remains sterile, preventing the epithelial cells from direct interaction with gut microbes. In the healthy gut, the TJs are intact limiting migration of molecules through the intercellular space. Lower left depicts common TJ proteins and it connection to the zonulin level influencing the integrity of the tight junctions. Right side depicts dysbiosis where the mucus turnover is reduced and epithelial cells is exposed to intestinal microbes and their biochemical action that potentially of weekenes the TJ interaction between the epithelial cells. During this induced increased intestinal permeability (IP), the gap between adjacent cells is widened, casusing this increased passage of substances. The figure shows how lactulose, L (Galβ1-4Fruc), yellow circle-green pentagon) and mannitol, M (Man-ol, green circle) can be used for measuring increased IP. L are transported via a paracellular route, enhanced by increased IP, while M are transported mainly via a transcellular route, especially when IP is normal [5]. Figure produced using Biorender (www.biorender.com)

The intestinal barrier is a complex structure that covers a surface of about 400 m^2^ and acts as a selective interface between the external environment and the internal body tissues. This selective barrier restricts the movement of potentially harmful microbes and antigens whilst allowing water and electrolytes through [1]. The term intestinal barrier was first introduced by Cummings in 2004 to describe the intricate structure that acts a partition between external environment and the contents of the intestine [2]. In the large intestine, the epithelial surface is covered by mucus. This mucus acts as the body’s first line of defense against pathogens, preventing external molecules and bacteria from directly contacting with the epithelial cells in the gut lumen. The large intestine (colonic) mucus is separated into two layers, the outer and inner mucosal layers (Fig. 1) [3]. The less dense outer mucus layer is not in contact with the intestinal wall and is inhabited by commensal bacteria. This design helps to maintain the intestinal barrier integrity. The commensal bacteria in the outer layer compete with the pathogenic species for space and resources, limiting pathogenic growth. In contrast, the inner mucus layer is dense, sterile and impervious to bacteria and contains antimicrobial properties [3, 4]. Underneath the mucus, the innermost layer of the large intestine is composed of epithelium, which provides a physical barrier restricting the translocation of luminal contents. The epithelium consists of specialized cells, such as enterocytes responsible for water absorption, and mucus-producing goblet cells, which secrete mucus. Furthermore, it is also composed of other cell types such as enteroendocrine cells that produce various hormones [2].

Intestinal permeability (IP) refers to the control of material passing through the intestinal wall. The intestinal wall is made up of a layer of cells that are tightly joined together by protein complexes as tight junctions (TJs). These TJs play an important role in regulating the passage of substances, such as nutrients and waste products, from the lumen to the bloodstream. The IP is influenced by a variety of factors, including the size and solubility of the molecule, as well as the regulation of proteins in the TJs. When the TJs are disrupted or damaged, it can lead to increased IP, allowing larger molecules, toxins, and even microorganisms to pass through the intestinal wall (via paracellular transport) into the bloodstream [2] (Fig. 1).

Zonulin is one of the proteins that has been identified as a regulator of intestinal TJs. Increased permeability has been shown to be associated with higher zonulin levels. Extracellular zonulin stimulates EGF receptor signalling and protease activated receptor 2 (PAR-2) disassembling the TJs via interaction between actin and TJ proteins [6]. This results in the loosening of TJs and enhancing IP [7]. Glucagon-like peptide 2 (GLP-2) is also used to assess IP. GLP-2 is a hormone that is primarily secreted by the enteroendocrine L-type cells. It is an essential component that aids intestinal growth and its maintenance, nutrient absorption, and maintenance of mucosal barrier function [8].

### Carbohydrate derivatives to measure IP

The use of carbohydrate derivatives as non-invasive markers to test the degree of IP in humans was first introduced by Menzies *et al*., 50 years ago [9] and are still the most commonly used IP markers (Table 1). The use of sugar derivatives as markers has many advantages in that the molecules are very polar, stable at physiological environment, not prone to metabolism, nontoxic, have a relatively short lifetime, and they are selected so they are not produced endogenously. When used for testing the degree of IP, they must be selected against their utility as food or supplement, as many of them are used as sweeteners. The doses used in the IP tests should not introduce side effects and should be easily detectable, for instance, in urine by LC–MS. The molecules can permeate through the postulated transcellular or paracellular pathways (Fig. 1) using polar- or non-polar dependent transmission through the epithelial layer [10]. The pathway for a particular molecule through gut epithelial layers is determined by its size, its interaction with intra-or extra-cellular components like the TJs, and the integrity of the gut epithelia and mucus layer. The assumption is that eventually the derivative will reach the blood stream, before excretion and the level measured in urine. Baseline excretion of these molecules (if any) prior to administration is important to measure and could display large individual variations dependent on factors such as diet, medications, health condition, genetics, geographical location and extraction methods. To add to the complexity of measuring IP, the five sugar molecule markers vary considerably when it comes to their physiochemical properties (Table 1).

**Table 1:**
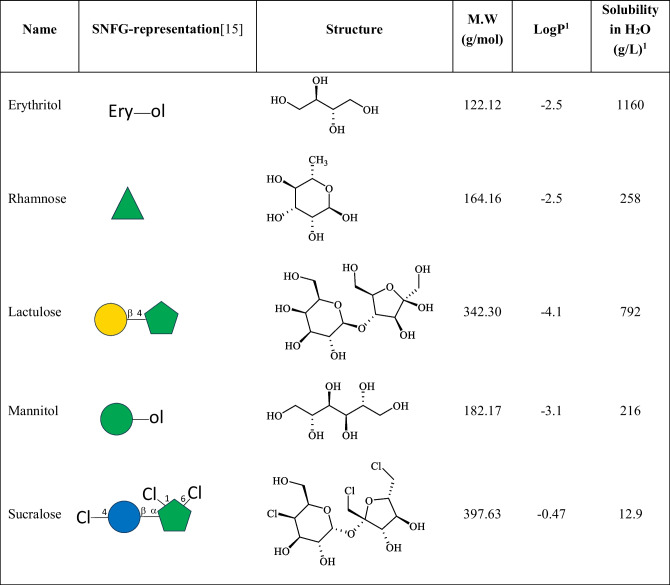
Common carbohydrate derivatives used for monitoring IP

^1^Solubility and logP values are taken from https://go.drugbank.com/

Measurements of the ratio of the sugars lactulose (L)/mannitol (M) in urine samples became the gold standard used to evaluate overall IP of the small intestine [11]. L and M do not only differ in their molecular weights, polarity, and solubility in water, but also in their three-dimensional structure. L has a molecular mass almost twice as large as M and L has a logP of -4.1, while M is one order of magnitude less polar (Table 1). M is a linear six carbon sugar alcohol derived from mannose. It is often founds as a sweetener but it also has medical usages [12]. M is absorbed in the intestine and is primarily excreted unchanged via the urine with elimination half-life of 4.7 h following oral administration [13]. Hence, M has the ability to cross the intestinal barrier via diffusion, independently if the barrier is intact, damaged or dysfunctional (Fig. 1). Lactulose (L) (Galβ1-4Fruc) is a bicyclic voluminous reducing disaccharide. L is not absorbed in the small intestine (Fig. 1) but unlike M, L will be readily fermented in colon by its microbiota [14]. M’s and L’s *in vivo*-conformations, in addition to molecular weight and polarity/solubility, would provide information of how L is able to cross the intestinal barrier and if it is only via the suggested (compromised) paracellular route [15].

Erythritol is a four sugar carbohydrate alcohol derived from reduction of erythrose and used as a sweetener. It is the smallest derivative of the five probes (Table 1) and has the highest solubility. Erythritol it is neither metabolized endogenously nor digested by the intestinal flora [16]. Rhamnose is a natural occurring six carbon deoxy-pyranose monosaccharide. It is regularly used to conduct the dual sugar permeability test as it is also assumed to be an inert non-metabolized sugar, However, it has been found to be fermented by colonic bacteria into rhamnulose [17]. Furthermore, oral administration of rhamnose has been suggested to be partially metabolized and excreted in urine as rhamnitol [18]. Sucralose is a synthetic organochlorine sweetener produced by chlorination of sucrose. It has the largest mass and highest logP of the carbohydrate derivatives in Table 1 and hence it displays the lowest solubility in water. Sucralose is not hydrolyzed in the intestinal lumen, is poorly absorbed and is excreted largely unchanged in the faeces [19].

Among the larger derivatives, L is the most polar. L is poorly absorbed in the gastro-intestinal tract and is broken down in the colon by colonic bacteria primarily to lactic acid, and to lower level, fermented into formic and acetic acid. Only low amounts of orally administered L is able to reach the blood in an uncompromised intestine. Urinary excretion has been determined to be 3% or less and is essentially complete within 24 h [20, 21]. Sucralose resembles L, but differ in its three-dimensional structure explaining the difference in polarity and solubility between the molecules. Due to its degradation by colonic bacteria, L is preferably used for IP measurement in small intestine, while the inert sucralose is used for colonic IP. The large difference in LogP between the molecules means that their paracellular IP pathway could differ.

Other probes include Chromium-labelled EDTA (^51^Cr-EDTA) and polyethylene glycols (PEG) that both work on a similar size exclusion principle for paracellular/transcellular transport. Biomarkers, such as the already mentioned zonulin and GLP-2, can also be used to assess IP. Elevated levels of zonulin peptide were found to be associated with increased IP, whereas elevated GLP-2 levels are associated with improved gut barrier function [22].

### IP and diseases

IP can be influenced by various factors, including changes in the gut microbiota, chronic stress, use of non-steroidal anti-inflammatory drugs (NSAIDs), alcohol consumption, infections, and certain medical conditions. Additionally, lifestyle and dietary habits, such as consuming a Western-style diet that is high in fat and carbohydrates, can also contribute to an increased IP [2].

Numerous chronic diseases, including inflammatory bowel disease (IBD, Crohn’s disase and ulcerative colitis), irritable bowel syndrome (IBS), diabetes, metabolic syndrome and autoimmune disorders, like Coeliac Disease (CD) are linked to IP [6, 23]. The link between IP, TJ and various disorders is known to exist, and the mechanisms underlying these associations are imerging [6, 24]. In this systematic review, we identified clinical studies investigating IP in diabetes (type 1 and 2) patients [25, 26] and CD patients [1]. CD is a disease that triggers an autoimmune response to the ingestion of gluten, and gluten-related proteins [27], which results in the attack of host tissue, harming the lining of the small intestine and dysfunction of the intestinal barrier (Fig. 1) [1]. Studies conducted on animals and humans indicate that changes in colonization during early life may enhance an individual's vulnerability to food sensitivities and chronic inflammatory illnesses [28, 29]. Recently, additional factors were suggested for CD developments, including gut microbiota dysbiosis and early disruption of the gut barrier [1, 30]. An increased IP has been demonstrated in patients with active CD [31, 32]. One study conducted on patients with CD, along with their relatives, healthy controls and individuals with aspecific gastrointestinal symptoms, demonstrated that in relatives of CD patients the L/M ratio was significantly lower than in the CD patient group [33]. This supports the theory that in CD, the intestinal barrier abnormalities correlate and act as a precursor to inflammation.

In metabolic disorders such as obesity, increased IP has been found in animal models and clinical studies [34]. Clinical studies have shown reduction in IP following a weight reduction treatment in individuals with obesity [35]. Weight reduction treatment was associated with significant improvements in the alpha-diversity of the gut's microbiota and the intestinal permeability. Another study demonstrated an increase in the IP of individuals with type-2 diabetes mellitus [36].

### Microbiota

The human gastrointestinal (GI) tract inhabits a vast and diverse community of microorganisms, including bacteria, archaea, fungi, viruses and other eukaryotes. This community of anaerobic microorganisms are collectively known as the “gut microbiota” [37]. The gut microbiota plays a crucial role in human health by providing metabolic, immunologic and protective functions through the symbiotic relationship with their host [38]. The gut microbiota consist of 10^10^–10^12^ live microorganisms per gram of human colon content [39–41]. The bacterial component of the gut microbiota in humans consists largely of two dominated phyla, Firmicutes and Bacteroidetes, which contribute to ~ 90% of the bacterial species [38, 42, 43].

The microbial colonization starts prepartum and proceeds to develop all the way from infancy to adulthood [44]. Recent studies suggest that the development of human microbiota starts already during early pregnancy inside the uterus. Some of the bacteria in the placenta are also found in mother's oral cavity and gut, indicative that that the bloodstream are able to transmit bacteria from mother’s mucosal tissue to the fetus [45]**.** The colonization of the intestines starts after birth and is affected by various factors like the mode of delivery [46, 47], diet [48], probiotic supplementation [49], the use of antibiotics and the maternal microbiome community during pregnancy [50, 51]. Infants born vaginally acquire microbiota resembling that of their mother's vagina, while infants born via cesarean section acquire microbial communities typically found on human skin. The acquisition of microbiota continues over the first few years of life, and an infant's GI tract microbiome begins to resemble that of an adult as early as 1 year old [7].

An altered composition or functionality of the gut microbiota is also known as “dysbiosis”. It is an imbalance of gut bacteria that has been associated with the pathogenesis of several inflammatory diseases and various infections [38, 52, 53]. This association may be attributed to the potential increase in IP. Animal studies have shown that a dysbiotic gut microbiota generates high levels of lipopolysaccharides (LPS), a structural part of gram-negative bacteria cell walls and endotoxin, which leads to inflammation of the intestinal mucosa, loss of TJ integrity between epithelial cells, and increased IP [25]. Increased levels of circulating LPS and changes in the immune response caused by an overgrowth of specific pathogens trigger systemic inflammation and IP [54].

Several factors, such as host physiology (*e.g*., age, disease, stress), genetics and environmental conditions (such as living conditions and medication use) can influence the GI microbiota [55]. Diet is increasingly recognized as an important factor that affects the composition and metabolic function of the GI microbiota. Western-style diet, which is characterized by its high fat, high sugar and low fiber content may lead to changes in the composition of gut microbiota, which causes an increased IP and elevated endotoxin concentration. Consumption of certain dietary ingredients, such as prebiotics, is one way to change and regulate the microbiota [56].

### Prebiotics

Prebiotics are non-digestible food ingredients that promote the growth and activity of beneficial bacteria in the gut, such as bifidobacteria and lactobacilli. These ingrediants typically resist digestion in the upper GI tract and are not broken down by the human digestive enzymes, so they reach the colon intact where they serve as a substrate for fermentation by the gut microbiota. Prebiotics are predominantly found in natural food such as onions, garlic, leeks, asparagus, bananas, oats, barley, wheat bran, and legumes [39].

Prebiotics were first described as “*a non-digestible food ingredient that beneficially affects the host by selectively stimulating the growth and/or activity of one or a limited number of bacteria in the colon, and thus improving the host health*”, by Glenn Gibson and Marcel Roberfroid in 1995. According to this definition, only a few compounds of the carbohydrate group, such as short and long chain fructans, lactulose, and galactooligosaccharide (GOS), could be classified as prebiotics [39].

In 2008, the International Scientific Association of Probiotics and Prebiotics (ISAPP) updated the definition of prebiotics to be *"a substrate that is selectively utilized by host microorganisms conferring a health benefit"* [39]. After the revision of the definition, specific criteria are employed to determine whether a compound can be classified as a prebiotic, which include:

(i) being resistant to acidic stomach pH, the substrate must not be hydrolysed or absorbed in the stomach or small intestine;
(ii) being fermentable by intestinal microbiota;
(iii) selectively stimulating the growth and/or activity of intestinal bacteria, which should induce beneficial luminal/systemic effects within the host [57]

The updated definition of prebiotics now includes the possibility of considering additional substances like linoleic acid, polyunsaturated fatty acids, phenolics, and phytochemicals as potential candidates for prebiotics [56]. Glyco-related prebiotic glycans can be divided into four main groups: polyols, oligosaccharides (including also human milk oligosaccharides (HMOs), Glycosaminoglycans (GAGs) and dietary fibres (Table 2).

**Table 2:**
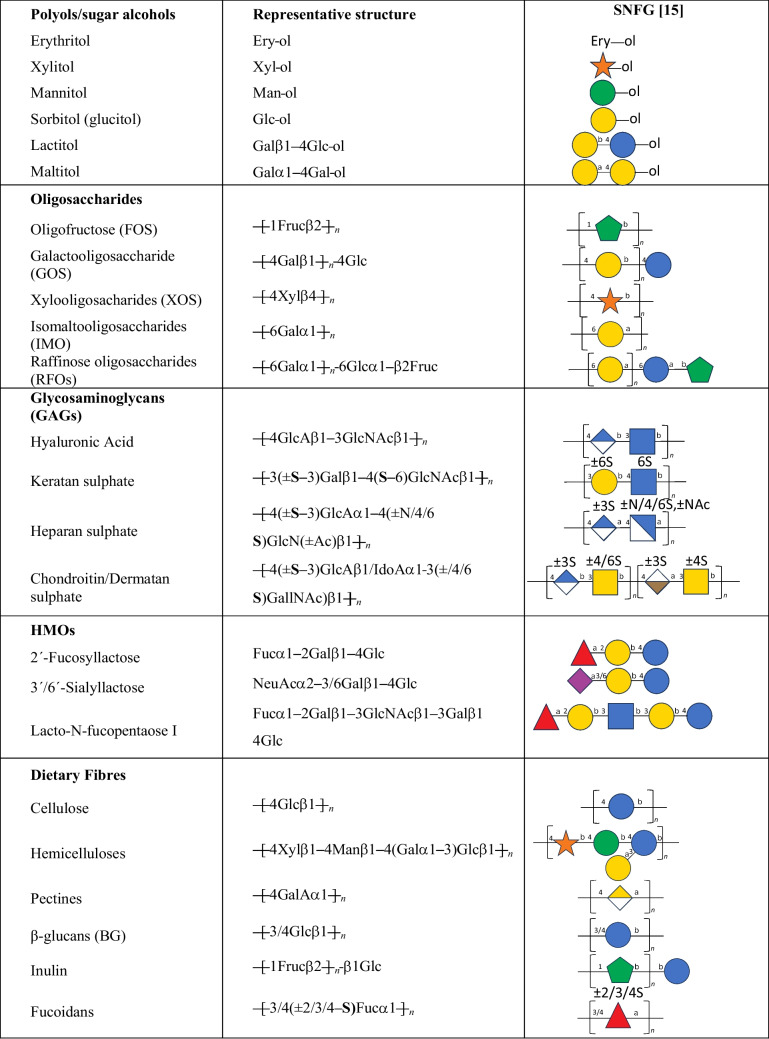
Types of glycobased prebiotics

### Polyols

Polyols are a specific group of sugar alcohols that are produced through the catalytic hydrogenation of carbohydrates. These compounds are made up of monosaccharide or di-/oligo-saccharides in which the hemiacetal or hemiketal functional group is reduced into alcohol. Most polyols are naturally occurring substances as they are found in certain fruits, vegetables, and mushrooms [58]. Additionally, polyols are also commonly used as sugar-free sweeteners in products such as candies, and beverages. Some examples of polyols include erythritol, lactitol, maltitol, mannitol (M), sorbitol, and xylitol [56] (Table 2).

### Oligosaccharides

Oligosaccharides are carbohydrates that are made up of a few monosaccharide units (typically 3–10). Some examples of oligosaccharides include oligofructose (FOS), which are oligomers of fructose monosaccharides; GOS, composed of galactose molecules and xylooligosaccharides (XOS), oligomers of xylose molecules [56].

### Glycosaminoglycans (GAGs)

GAGs are linear amino sugar-containing polysaccharides comprised of repeating disaccharides (Table 2) and are components of meat and dairy products [59], providing a prebiotic source throughout human life. Apart from hyaluronic acid, all GAGs are sulphated at various positions within the chains.

### Human milk oligosaccharides (HMOs)

Human milk oligosaccharides are complex sugars that are naturally found in human breast milk. This milk is a highly intricate substance that provides the newborn with tailored nourishment and contains various components that stimulate the growth of the intestine and contribute to the development of mucosal defenses. HMOs are thought to have multiple biological effects, with significant prebiotic roles. Human milk not only delivers nutrients but also includes hormones, growth factors, immunoglobulins, cytokines, and enzymes. The concentration of HMOs varies among individuals, but generally, HMOs make up to 5–15 g/l of breast milk [60, 61].

Distinctive HMOs are synthesized within the mammary gland, where specific glycosyltransferases add several monosaccharides to a lactose core. Lactose-based HMOs consist of five fundamental monosaccharides: glucose (Glc, galactose (Gal), *N*-acetylglucosamine (GlcNAc, fucose (Fuc), and sialic acid (NeuAc) [60]. Some examples of common lactose-based HMOs include, 2'-Fucosyllactose, 3'-Sialyllactose, 6'-Sialyllactose and Lacto-*N*-fucopentaose (Table 2). As mentioned earlier, milk also contains GAGs. The main GAG types in human and bovine milk are CS and HS, with smaller quantities of dermatan sulphate and hyaluronic acid also present [62].

In breastfed infants the intestinal microbiome is rich in bifidobacteria, which are know to deflect pathogens. Therefore, HMOs (including milk-derived GAGs) are thought to promote the development of a bifidogenic-dominated microbiome. Various HMOs have structural similarities with mucin glycans found on the intestinal epithelia of infants, which are used by various pathogens as receptors. Free HMOs may also act directly on pathogens by blocking pathogenic proteins from attaching to intestinal epithelial cells in breast-fed infants [60, 63–67].

Many bifidobacteria possess specific enzymes for catabolism and metabolism of carbohydrates [68], suggesting that HMOs and GAGs provide a nutrient resource for bifidobacteria that can shape their growth, activity and niche colonization [69, 70].

### Dietary fibre

Although the precise definition of Dietary Fibre (DF) is still evolving, DF is described as *“Carbohydrate polymers with ten or more monomeric units, which are not hydrolyzed by the endogenous enzymes in the human small intestine”* according to the Codex Alimentarius Commission. By this definition, DF is mostly polysaccharides, although some countries have oligosaccharides categorized as DF. Some of the DF include cellulose, hemicellulose, inulin, pectines, fucoidans [71] and β-glucans [72]. Inulin is also one of the prebiotics that is widely used and can be classified either as an oligosaccharide or a polysaccharide depending on its Degree of Polymerization (DP), which is a term used to describe a number of repeating monomer (Fructose, Fruc) units. Inulin belongs to the fructans subgroup of carbohydrates, and it differs from FOS mainly because of the DP; inulin contains DP up to 60 units whereas FOS generally contains less than 10 [73].

### The role of prebiotics in the gut

**Fig. 2:**
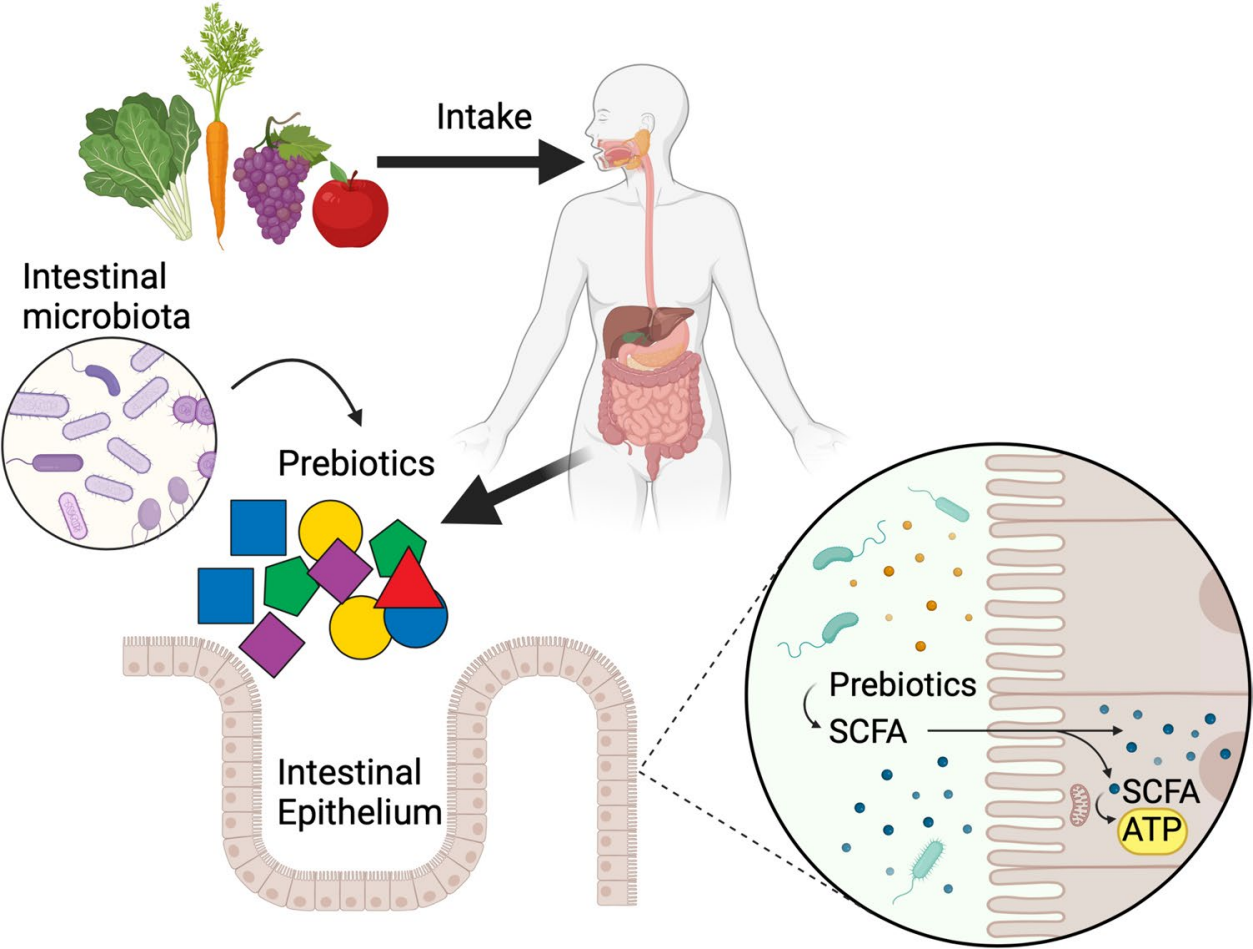
Potential effects of prebiotic intake in human gut. Food containing prebiotic oligosaccharides reach the colon, where the microbiota converts the prebiotic into short chain fatty acids (SCFA), that intestinal cells can use to generate energy. Figured produced using Biorender (www.biorender.com)

The consumption of prebiotics improves human health by controlling the imbalance of the gut microbiota and promoting the proliferation of helpful bacteria, such as Lactobacillus and Bifidobacterium, which impedes the colonization of harmful bacteria as well as reducing the risk of development of various diseases including colon cancer, irritable bowel syndrome, type 2 diabetes mellitus, and obesity [39]. The main bacterial fermentative end products of complex carbohydrates are Short Chain Fatty Acids (SCFAs), namely acetate, propionate, and butyrate, and gases (H_2_, and CO_2_) [56] (Fig. 2).

## Objective

The objective of this study was to evaluate the effect of prebiotic intervention on IP in humans by comprehensive analysis of relevant published studies, with a focus on randomized controlled trials (RCTs). Based on the selected studies, we identified that the molecular methods used to asse IP were:

1. Excretion of adminstered probes such as Lactulose (L) and Mannitol (M).
2. Level of indigenous biomarkers such as serum and fecal zonulin levels and serum GLP-2 levels.
3. Changes in gut microbiota composition.

Furthermore, the review aimed to identify the current evidence base, to analyze the quality of the recent studies about the subject, and to identify the overall effect of prebiotic supplementation on IP.

## Methods

A systematic review was performed using the following eligibility criteria:

To be eligible for inclusion studies must have met the following criteria,

- **Population:** Human studies
- **Intervention:** Any non-digestible carbohydrate-based prebiotics administration with the explicit intention to see the changes in the intestinal permeability
- **Comparisons:** Non-intervention
- **Outcome:** The effect of prebiotic intake on intestinal permeability

### Search strategy

Initially, a preliminary exploration was conducted on the PubMed database and Google Scholar. Then, a search was conducted on the databases PubMed and Trip, utilizing various combinations of keywords like "prebiotics", "carbohydrates", "glycans", "intestinal permeability", and "leaky gut" termed with the Boolean Operator such as, “AND” (Table 3).

**Table 3:** Search keywords, the number of hits and in both PubMed and Trip database

Search Combinations	PubMed	Trip
(prebiotics) AND (intestinal permeability)	271 results	152 results
(prebiotics) AND (leaky gut)	54 results	21 results
((prebiotics) AND (carbohydrates)) AND (intestinal permeability)	159 results	36 results
(prebiotics) AND (carbohydrates) AND (leaky gut)	20 results	5 results
((prebiotics) AND (glycans)) AND (intestinal permeability)	149 results	3 results
(prebiotics) AND (glycans) AND (leaky gut)	18 results	1 result

The results obtained from the search were initially screened by examining article titles and abstracts. After exclusion of articles that were not relevant to the subject area, further narrowing was done by reading the full text of the identified articles. Studies that did not establish a connection between prebiotics and IP were then also excluded. Full texts of potentially relevant articles were retrieved and assessed. The time period considered for article inclusion was from 2010 to 2023.

### Identification of studies

**Fig. 3:**
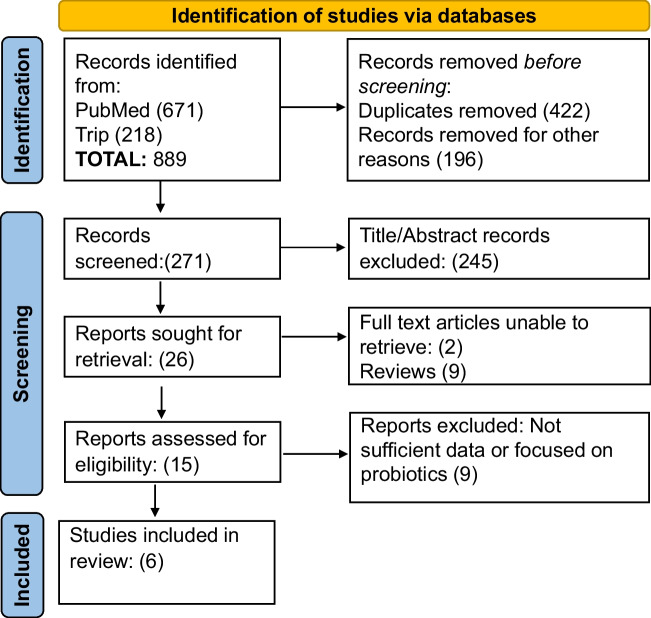
Study selection flow diagram

A total number of 889 articles were identified but only 6 met all the selection criteria. (Fig. 3) During the first selection, 422 of the articles were excluded as they were duplicates and another 196 were excluded because they were either ongoing clinical trials, ongoing systematic review, or guidelines. During the screening phase of the remaning 271 articles (Supplemenary Material 1), 245 of the articles were excluded based on their titles, abstract and respective keywords. All the studies that were performed on animals and *in vitro* were excluded. Of the remaining 26 articles, 9 of them were reviews and were hence excluded. Two articles were excluded as it was not possible to retrieve the full text (not available as open access or included in the library service in our institute). After reading the 16 full text articles, the additional 9 were excluded because they either provided insufficient results or because the article focused on probiotics rather than prebiotics. The summary of the 6 remaining articles is provided in Table 4.

**Table 4:** Summary of all the articles, including the type of study, population, interventions and the outcome

Study	Population	Intervention	Influence	Ref
(Study 1) Determine the effect of enteral supplementation of GOS/FOS/acidic oligosaccharides on IP in the first week of life in preterm infants	113 Infants with a gestational age (GA) < 32 weeks and/or birth weight (BW) < 1500 g	Enteral supplementation of 80% GOS/FOS with 20% non-human milk acidic oligosaccharides or a placebo of maltodextrin (MD) for 1 week	The prebiotic group had a decrease in L/M ratio, but it was not statistically significant	[74]
(Study 2) Determine IP and the circulating levels of zonulin and in healthy young volunteers using inulin-enriched pasta	20 healthy male volunteers with an average age of 18.8 ± 0.7 years	Inulin enriched pasta or control pasta, two 5-week periods of study separated by an 8-week washout period and 2-week run-in period	L/M excretion ratio was significantly decreased between baseline, control pasta and the intervention. P = 0.012	[8]
(Study 3) Determine the host–microbiome interactions in human type 2 diabetes (T2D) following prebiotic (GOS) intake	29 Men with well-controlled T2D aged 42–65 years	Prebiotic fibre (GOS) or placebo (MD) supplementation for 12 weeks	Prebiotic supplementation had no significant effect on IP, when compared to placebo	[26]
(Study 4) Determine the effect of prebiotic on microbiota, IP, and glycemic control in children with type 1 diabetes (T1D)	38 children aged 8 to 17 years that had T1D for at least 1 year	8 g of FOS enriched inulin every day or 3.3 g of MD orally every day for 12 weeks	The prebiotic group had a decrease in IP although this difference did not reach statistical significance	[25]
(Study 5) Determine IP in children with CD after the administration of oligofructose-enriched inulin into a gluten-free diet	34 children aged with diagnosed CD; age not specified	10 g of FOS-enriched inulin per day or a placebo (MD) per day for twelve weeks	No significant difference was observed on IP for the experimental groups at enrollment (T0) and after the intervention (T1)	[1]
(Study 6) Determine the effects of dietary fibers on acute indomethacin induced intestinal hyperpermeability in the elderly	49 Individuals aged 65 years and older	daily supplementation (12 g/day) of oat -glucan, arabinoxylan (AX) or placebo (MD) for six weeks	no significant differences were observed in the two treatment groups when compared to placebo	[75]

## Results

The folowing six studies (see Table 4) were analysed and compared:

### Effects of prebiotics supplementation on preterm infants (Westerbeek *et al*. 2011, Study 1)[74]

Research was conducted to determine the effect of enteral supplementation of GOS/FOS/acidic non-human milk oligosaccharides on intestinal permeability as measured with the sugar absorption test in the first week of life in preterm infants. For this purpose, infants with a gestational age < 32 weeks and/or birth weight < 1500 g were studied. The infants were randomly allocated after birth to receive either an enteral supplementation of a prebiotic mixture of 80% GOS/FOS and 20% acidic non-human milk oligosaccharides or a placebo mixture of Maltodextrin (MD). The preterm infants were studied for a week and were divided into two groups, one group of 55 infants, who were given GOS/FOS/ acidic non-human milk oligosaccharides while another group of 58 infants were given a placebo. Intestinal permeability was studied by measuring the excreted L/M ratio in both groups between time points t = 0, 36 h after their birth, t = 1, postnatal day 4 and t = 2, postnatal day 7. L and M excretion were measured using gas chromatography. The study observed a decrease in L/M ratio, from t = 0 (0·21 v. 0·34), to t = 2 (0·06 v. 0·09) in prebiotic supplemented groups and placebo groups, respectively. Although both groups showed a decrease in L/M ratio, this decrease was not statistically significant. Therefore, the study found that the prebiotic supplement had no effect on the L/M ratio between t = 0 and t = 2.

The researchers then investigated the combined effect of different host and treatment factors, such as gestational age, birth weight, exclusive breastfeeding, and mixed feeding, on intestinal permeability in both groups. The significant results obtained revealed that increased body weight was associated with a decrease in intestinal permeability. Furthermore, infants who were exclusively breastfed or received a mixture of breast milk and formula during the first week of life had lower L/M ratios than infants that were exclusively formula-fed [74].

### Use of inulin enriched pasta on IP in healthy individuals (Russo *et al*. 2011, Study 2)[8]

This study was conducted to find the influence in administration of inulin-enriched pasta to healthy young volunteers and their effect on IP. The study included 20 male volunteers who were in good health and completed the study. Their mean age was 18.8 ± 0.7 years. The study design was a randomized, double-blind crossover with a baseline evaluation and two 5-week periods of study separated by an 8-week washout period. There was also a 2-week run-in period to ensure that all participants began at similar levels of the intervention. The intervention with inulin enriched pasta contained 11% inulin from chicory and the control pasta was made of 100% durum wheat semolina.

L and M probes were used to measure IP by calculating the urinary L/M ratio using high-performance anion-exchange chromatography. Serum and fecal zonulin levels were also measured using enzyme-linked immunosorbent assay (ELISA).

The study showed that the inulin-enriched pasta diet had improved IP as it provided the lowest L/M excretion ratio of 0.03 which was significantly different (P = 0.012) compared to the baseline (L/M ratio 0.05) and control pasta (L/M ratio 0.05). L excretion ratio was also found to have decreased significantly (0.45%) when compared to the baseline (0.71%) and control pasta (0.75%) diets while the M levels stayed the same.

Regarding the zonulin serum levels, when compared to baseline values (5.24 ng/mL) and control pasta group (5.59 ng/ml), the group that had pasta enriched with inulin showed descreased zonulin (3.63 ng/ml). The zonulin levels in the inulin-enriched pasta group (0.20 g/g feces) were the same as those in the baseline group (0.19 g/g feces) and the control pasta group (0.19 g/g).

The GLP-2 content (median and the range) in blood samples obtained after overnight fasting was higher and significant (P = 0.004) in the inulin-enriched pasta group (5.10 ng/ml; 4.38–8.05 ng/ml) compared with the baseline (4.85 ng/ml; 3.95–6.40 ng/ml) and the control pasta groups (4.99 ng/ml; 3.97–6.65 ng/ml) [8].

### Effects of prebiotic supplementation in adults with Type 2 Diabetes (T2D) (Pedersen *et al*. 2016), Study 3)[26]

This study was conducted to determine the effects of prebiotic supplementation on intestinal bacteria and IP in adult patients with T2D. Men with well-controlled T2D aged 42–65 years were recruited, and a randomized, double-blind, placebo-controlled parallel study was conducted. Women were excluded from the study as the radioactive ^51^Cr-EDTA is believed to have influence on the menstrual cycle. Patients were randomized to either prebiotic group receiving, GOS or placebo supplementation (MD) for 12 weeks. Urinary excretion of orally administered ^51^Cr-EDTA was used to measure the IP over a 24-h period. ^51^Cr-EDTA was used as it is not easily degraded by colonic bacteria and because of its stability in the colonic luminal environment, and this facilitates its detection in the urine. Blood samples were also taken to study C-peptide levels as well as glycosylated hemoglobin (A1C) levels. Regarding the gut microbiota, total bacterial group was quantified using real time Polymerase chain reaction, PCR (qPCR).

After the study, it was possible to conclude that prebiotic supplementation had no significant effect on IP, as measured by urinary recovery of ^51^Cr-EDTA when compared with placebo. No significant difference was also found in the A1C and C-peptide levels (diabetes markers). Diversity (α and β) were also studied, where α-diversity describes the microbial species diversity within the sample, and in context of this study it refers to the diversity of microbial species within each group, prebiotic and placebo. Whereas, β-diversity describes the taxonomical, meaning the differences in the gut microbial species composition differences between thee prebiotic and placebo group [76]. No significant difference in β-diversity, evenness (the relative abundance of species) and richness (the number of species per sample) was found in prebiotic group when compared with placebo. However, the study did find that within the prebiotic group, the bacterial α-diversity and richness were increased. The gut microbiota composition remained largely unchanged after the intervention whereas the PCR results revealed that the Bifidobacterium levels increased in both groups; with a greater change observed in the prebiotic group, which approached significance (P = 0.0582) [26].

### Effects of prebiotics supplementation on children with Type 1 Diabetes (T1D) (Ho *et al*. 2019), Study 4)[25]

The research was conducted to determine the effect of prebiotic supplementation to alter gut microbiota and IP in children with T1D and assess whether such changes could improve glycemic control as well as examine the differences in gut microbiota and IP. Children aged 8 to 17 years that had T1D for at least 1 year were studied. Participants were randomly selected to the prebiotic or placebo group. The placebo group received 3.3 g of Maltodextrin (MD) orally each day whereas, the prebiotic group received 8 g of FOS enriched inulin. The intervention in total lasted for a total of 3 months of intervention followed by a 3-month washout period with no intervention. IP was assessed at baseline, 3 months, and 6 months by measuring the L/M ratio recovered in the urine after oral ingestion of the two sugars. For gut microbiota profiling, stool samples were collected at baseline, 3 months, and 6 months and Bacterial community composition was determined by 16S rRNA sequencing and absolute abundance of *Bifidobacterium spp* was quantified with PCR. To determine whether the use of prebiotics has any influence on diabetic control, blood samples were also drawn during the baseline, 3 months, and 6 months after the intervention where C-peptide and A1C were also studied. The study observed that after 3 months of FOS-enriched inulin treatment, the prebiotic group had a decrease (-0.005) in L/M ratio compared to the baseline values, indicating the decrease in intestinal permeability although this difference did not reach statistical significance (P = 0.076).

In terms of metabolic changes, there was no impact on A1C levels observed in the group that received prebiotics during the 3-month intervention, as compared to the placebo group. However, after the prebiotic intervention was completed, a noteworthy association was observed between A1C and the L/M ratio (P < 0.01). Regarding the C-peptide levels, the prebiotic group demonstrated a noteworthy increase (P = 0.029). After the 6 months, washout period, the study observed a significant correlation between A1C and C-peptide (P < 0.01) in the prebiotic group.

When it comes to the gut microbiota, the study found that the use of prebiotics had bifidogenic effects, as indicated by a higher relative abundance of *Bifidobacterium spp*. at 3 months compared to the placebo group, but this increase did not remain after 6 months (washout period). There where also a negative correlation between relative abundance of the bacterial genus, *Terrisporobacter* and C-peptide after 3 but not after 6 month.

The study found that the α -diversity of the microbiota was slightly decreased in the prebiotic group compared to the control group, as measured by the Shannon index. On the other hand, the β -diversity, overall microbial community was significantly different between the prebiotic and control group [25].

### Use of Prebiotics regarding IP in Children with CD (Drabińska *et al*. 2020, Study 5) [1]

This study was conducted to evaluate the effect of twelve-week supplementation of a prebiotic FOS-enriched inulin (10 g per day) with gluten-free diet on the IP in children with CD.

Thirty children with diagnosed CD were studied in a randomized, placebo-controlled clinical trial. The age of these children was not disclosed in the study. These participants were randomized either to a prebiotic group called Synergy 1, (receiving 10 g of FOS-enriched inulin per day) or a placebo (MD) for twelve weeks. IP was measured using L/M ratio which was quantified by GC–MS. The analysis of plasma zonulin and GLP-2 levels were performed using a commercial ELISA Kit.

At the beginning of the study and after the intervention, there was no significant difference observed between the experimental and the placebo group. The values of L/M did decrease both in prebiotic (0.060 vs. 0.054) and placebo (0.063 vs. 0.056) after the intervention but these changes were not statistically significant. The study also evaluated the L and M excretion separately. In case of M, after the intervention, there was a significant increase in the mean excretion in the Synergy 1 group as it reached 20.77 ± 12.87% from the baseline value of 18.08 ± 13.86%. The placebo group meanwhile showed a decrease in M excretion for most participants from 24.59 ± 14.79% to 17.14 ± 6.15%. Regarding the L excretion after the intervention, there was a slight increase in its excretion in the Synergy 1 group as it reached 1.22 ± 0.93% from 1.07 ± 0.62%, but this was not statistically significant. In the placebo group there was a slight and non-significant decrease in the excretion as it reached 1.29 ± 0.84% from 1.89 ± 1.82%. As for the plasma zonulin level, it was increased after the twelve-week intervention, in both Synergy 1 group (28.66 ng/ml to 36.26 ng/ml) and in Placebo group (17.38 ng/ml to 28.82 ng/ml) respectively. GLP-2 levels on the other hand, no significant increase was observed in both groups [1].

### Effects of dietary fibers on acute indomethacin-induced intestinal hyperpermeability in the elderly (Ganda *et al*. 2020, Study 6)[75]

This study was conducted to investigate the potential effects of dietary fibers oat β-glucan (OBG) and wheat arabinoxylan (AX) in IP and its effect to counteract acute non-steroid anti-inflammatory drugs, NSAIDs, (indomethacin) induced hyperpermeability in the elderly population.

For this purpose, a Randomized Placebo Controlled Clinical trial was conducted where healthy individuals aged 65 or over were recruited. The participants were randomly allocated to groups receiving prebiotics, 12 g/day of OBG or 12 g/day of AX or group receiving placebo, 12 g/day of MD. The study was conducted in total of 9 weeks where the first three weeks were used to measure the baseline levels of the patients and the remaining six weeks were used to conduct the dietary intervention with prebiotic and placebo group as described earlier.

In this research, IP was studied in a different way where it was evaluated using a multi-sugar test with the following protocol: a solution of five sugars (sucrose, L, L-rhamnose, sucralose, and erythritol) was administered orally after overnight fasting. The urine samples were collected in two parts, where the first sample was collected within 0–5 h after the ingestion of the multi-sugar solution, while the second sample was collected between 5–24 h. The first sample was used to evaluate small intestinal IP using the L/Rhamnose ratio, while the second sample assessed colonic IP by measuring the Sucralose/Erythritol ratio.

To evaluate urinary excretion values of sugars post-indomethacin intervention, participants were given indomethacin doses followed by overnight fasting. In the next morning, second dose of indomethacin was given and an hour after that, the second multi-sugar test was conducted, and urine samples were taken in two samples as described above. Urinary sugar excretion was analyzed using LC–MS. For gut microbiota profiling, fecal samples were taken before and during the study (week 5) and bacterial community composition was determined by using a PCR that targets specifically the 16S rRNA gene.

Questionnaires were also taken before the start of the study and during the whole study to validate different symptoms such as GI discomfort, Stress, Anxiety and Depression and Quality of life. Different questionnaires were used during the study.

After the study was concluded, there was no significant difference observed in the two groups receiving prebiotics when compared to placebo before and after the indomethacin challenge. In the group receiving AX, the colonic IP decreased from 0.033 to 0.029 whereas the small intestinal IP decreased from 0.056 to 0.055 before and after the intervention respectively, although neither results were statistically significant. Meanwhile in the group receiving OBG, the small intestinal IP changed from 0.056 to 0.057 and the colonic IP remained constant. Regarding the effect of indomethacin before prebiotic treatment, it caused a significant increase in small intestinal IP in both groups, p < 0.05 and p < 0.001 in groups that received AX or OBG respectively, whereas only the group that received AX showed an increase in colonic IP, p < 0.01. Regarding the questionnaires, no significant differences were found regarding the symptoms observed during and before the study. In the gut microbiota, no difference was found in the overall β diversity between the intervention and placebo group, before and after the dietary intervention. Likewise, as assessed by the Shannon indices, which measure the α-diversity, no significant change was found within separate groups [75].

## Discussion

After review of the 6 articles, 5 out of 6 studies indicated an improvement in the IP after the prebiotic interventions, but only one with significance. In these studies, the type of prebiotics used were different from each other as no more than 2 studies used the same type of prebiotic. The prebiotic in use included GOS mixture (2 studies), Inulin (3 studies) and Fibers (OBG or AX) (1 study). While various types of prebiotics were utilized in the studies most, 5 out of 6 studies chose to utilize MD as the placebo.

The populations studied in those studies were also quite distinct from each other as the study examined a diverse range of populations, spanning from preterm infants (< 48 h after birth) to elderly individuals (≥ 65 years). This is quite beneficial as we can gain a better understanding of how a particular intervention or condition affects different groups of people. Not only that, but it also allows to identify interventions or treatments that are effective across different age groups, genders, or pathologies etc. However, it prevents us from drawing extended statistical conclusions of the use of prebiotics in a more defined area.

### Usage of different probes

In these 6 studies, various probes were used to study IP. Four of the 6 studies chose to use the L/M ratio to assess the IP whereas the remaining 2 studies used ^51^Cr-EDTA and a multisugar test. As L and M are two sugars not digestible by human enzymes and with different molecular sizes, they are absorbed differently in the intestine (Fig. 1). When administered orally, the sugars can cross the intestinal barrier by different paths and enter the bloodstream before excretion via urine. The use of these two sugars appears to be the gold standard presumably because of the ease of administration and measurement.

In the Study 6 conducted by Ganda *et al*. [75], different probes were used than the normal L and M to evaluate the IP. In addition to L, L- rhamnose, Sucralose and Erythritol were used. L and L-rhamnose were specifically used to assess small instetinal IP. L is a disaccharide that is not normally present in the small intestine and is poorly absorbed in the small intestine membrane whereas L-rhamnose is a monosaccharide that is rapidly absorbed. Sucralose and erythritol were used to study colonic IP. Sucralose is also a disaccharide and an artificial sweetener and erythritol is a polyol. Both molecules are poorly absorbed in the the small intestine, which is beneficial in this case. In the Study 3 by Pedersen *et al*. [26]_,_^51^Cr-EDTA was be used to measure IP as it is a molecule that remains stable throughout the GI tract and is not easily broken down by microbes, making it useful to study both small and colonic permeability. A 24-h urinary excretion test is therefore done when ^51^Cr-EDTA is used. As ^51^Cr-EDTA is a single probe, there is some potential for non-mucosal factors such as intestinal transit time, renal clearance and gastric emptying rate may significantly affect the recovery in urine.

### Implications of the review

In Study 1, conducted by Westerbeek *et al*. [74], the L/M ratio showed a decrease in both the prebiotic and placebo groups, but no significant difference between the two groups. When comparing the L and M values alone, it indicated that there were a decrease in the L values in both prebiotic and placebo groups, while in the same timeframe M levels remained relatively contant. The decrease of L excretion is in accordance with that L are degraded by the microbes in the colon. M is not degraded at the same rate and keeps diffusing through the intestinal barrier, throughout its passage of both the small and large intestine.

As the results were not statistically significant between the groups the researcher conducted additional analysis of factors that influenced the IP. During this analysis, it was found that the preterm infants who were exclusively breastfed or did a mix of breast- and formula feeding had a steeper decrease in their L/M ratio. This result suggests that during the first neonatal week, prebiotics did not provide any significant additional effects. Breast milk contains HMOs, which are beneficial for babies not only because support growth of healthy microbiota, but also provides protection against pathogens for the babies. The decrease in L/M ratio, which was statistically significant, suggests that breast milk feeding improves IP in preterm infants. As per study 1 by Westerbeek *et al*. [74], also prior research found that preterm infants who were breast-fed had a reduction in IP after 30 days of the study. Increase in body weight of the preterm infants also correlated with decrease in L/M ratio, although the reason exactly why this occurred remains unknown. The study 5, done by Drabińska *et al. *[1] explored the effect of prebiotic supplementation on patients with CD. FOS enriched inulin was the prebiotic used during the study. FOS is a short-chained carbohydrate, and it is fermented more in the proximal part of the colon whereas long-chained inulin is fermented slowly and more in the distal part of the colon. By combining both prebiotics, it can be proposed that the fermentation profile and the IP of whole colon can be studied [77].

Many studies have shown that individuals with CD have altered gut microbiota as well as increased IP. Currently, a gluten-free diet is the only known parameter to improve the condition. In a study done to evaluate long term effect of gluten-free diet intervention on individuals with CD, after a year the patients showed an improved IP [78]. Therefore, the addition of prebiotic as an adjunctive has been thought to be a promising intervention in this case. No significant difference in IP was found after the study as L/M ratio showed no significant improvement. When assessing two sugar probes separately, the increase in M excretion found in the prebiotic group when compared to placebo might have beneficial effect.

During CD, there is a decrease in mature small intestinal surface area, which leads to a reduction in the fractional excretion of small probes, such as M. Therefore, an increase in fractional M excretion can be associated as the first sign of improvement of the condition. Excretion of larger probes such as L can also be seen in individuals with CD, suggesting that, there might be alternate pathways that are accessible to L [79]. On contrary to M excretion, prebiotic intervention did not have any influence on L excretion. This way, we can assume that although overall L/M ratio did not show any significant change, the change in M excretion demonstrated an improvement of the condition in the prebiotic group.

In the study 4 by Ho *et al*. [25], the study design consisted of 3 months intervention period followed by a 3-month washout period where no intervention was taken. This step was taken to ensure that the treatment effect being measured is due to the intervention being studied and not influenced by the residual effects of a prior treatment or intervention. Although after 3 months of group supplementation no significant decrease in the L/M ratio in the prebiotic group was observed, a decrease was detected and it was close to the significance level (P = 0.076), when compared to the placebo group. This suggests that the use of prebiotics may have some positive effect on the IP.

A1C levels also did not show a significant decrease in study 4 by Ho *et al*. [25], but a positive correlation between A1C levels and the L/M ratio (P < 0.01) was found. A1C is a blood test that is done to measure the average blood sugar levels during the previous 3 months. According to this finding, a decrease in L/M ratio may contribute to a decrease in IP and better glucose control in individuals with type 2 diabetes. C-peptide levels on the other hand, were increased after 3 months. C-peptide is a type of peptide that is produced during the production of insulin, and it is typically secreted at a 1:1 molar ratio to insulin. Therefore, C-peptide levels can be studied to analyze the insulin production [80]. This finding demonstrates that the group who received prebiotics had an improvement in their β-cells function, as increased C-peptide levels indicate increased insulin production. This then leads to better glycemic control in case of T1D.

Similarly, the study 3 of T2D individuals conducted by Pedersen *et al. *[26], showed that, prebiotic intake did not show any improvement on IP when compared to placebo group. Previous studies have found that individuals with T2D have an increase in IP [81]. The lack of effect of the prebiotics in Study 3 may be due to the fewer numbers of people studied.

Inulin enriched pasta was used as prebiotic supplementation in study 2 by Russo *et al*. [8], where a significant decrease in IP levels between prebiotic and control group were found via the L/M ratio. This is the first study where a significant improvement in IP was found following a prebiotic intervention. Although the results are positive, it should be mentioned that the study was carried out on a group of healthy people. If the reported advantages of prebiotics apply in other situations, it would be crucial to perform additional research in people with compromised gut health. In spite of this, the positive findings of this study provide us with a promising sign of the potential advantages of prebiotic therapies for gut health.

During Study 6, the research conducted by Ganda *et al*. [75], baseline values of urinary sugar excretion were measured using multi sugar test in both groups that received prebiotics. A general baseline excretion ratio was measured, to see the "normal values" followed by the indomethacin intake and the subsequent sugar excretion. Indomethacin belongs to a group of drugs namely, non-steroidal anti-inflammatory drugs, NSAIDs, and are generally used because of its analgesic, anti-inflammatory and antipyretic properties and are also known to have some effect on IP. In an older study done on 12 rheumatoid arthritis patients before the intake of NSAIDs, all patients showed a normal excretion of ^51^Cr-EDTA. After the intervention with NSAIDs, 10 of the 12 patients showed a significant increase in IP, suggesting that the use of NSAIDs increase IP [82]. In study 6, similar findings could be seen as small intestinal permeability and colonic permeability increased significantly in the group receiving prebiotic AX whereas the group receiving OBG, only showed increased small intestinal permeability following an indomethacin intake when compared to their respective baseline values. This evaluation of small intestinal and colonic permeability is very beneficial as it gives us an overview of IP throughout the GI tract. After the study was conducted with prebiotics, baseline excretion levels were measured again, followed by the indomethacin intake and the subsequent measurement of sugar excretion. Prebiotic intake of dietary fibers such as AX or OBG did not have any effect on the acute intestinal hyperpermeability caused by the indomethacin intake as the small intestinal and colonic permeability did not decrease during pos-treatment with prebiotics when compared with the increased IP induced by indomethacin pre-treatment.

### Usage of Biomarkers to assess IP

Other than the use of probes, some studies also included the use of biomarkers to assess IP. Two of the studies measured zonulin and GLP-2 levels.

#### Zonulin

Regarding the zonulin level in study 2 by Russo *et al*. [8], the decrease in serum zonulin levels when compared to the control group and baseline value tells us that prebiotic interventions does affect the gut barrier by improving the tight junction function which leads to an improvement in the IP, as more zonulin levels is normally associated with tight junction deterioration and increased IP. Fecal zonulin did not show any difference when compared to the baseline value and control group. This may be due to various factors such as intestinal transit time or a potential bacterial degradation which may cause insufficient or reduced sensitivity. Zonulin level was measured also in study 5 by Drabińska *et al*. (2020) [1]. Plasma zonulin were found to be increased in both prebiotic and placebo groups. This result is inconsistent with the expected outcome as according to the slight decrease in L/M ratio found in the study, a slight decrease in zonulin levels was expected.

#### GLP-2

According to previous study by Cani *et al*. [83], which used a diabetic mice model to study the effects of prebiotic supplementation and its effect on IP, improved gut barrier function was found as well as an significantly increased GLP-2 levels after the treatment, suggesting that increase in GLP-2 values are associated with an improvement and preservation of the gut barrier function. GLP-2 values in study 3 by Russo *et al*. (2012) [8], showed a significant increase when compared to placebo, suggesting that during this time period, the use of prebiotics stimulated an improved gut barrier function. Contradictory to this study, in study 5 by Drabińska *et al*. (2020) [1], GLP-2 values did not show any significant increase when compared to placebo. This result is consistent with that neither L/M ratio nor zonulin levels showed any improvement in this study. Hence, it appears that in our metastudy, improved IP measured by L/M ratio, correlates with decreased zonulin and increased GLP-2 levels, indicating these factors are influencing each other.

### Evaluation of Gut microbiota

To evaluate the gut microbiota, all studies used stool samples and cell lysis method to extract DNA. Subsequently, a Polymerase chain reaction (PCR) primer targeting the 16S rRNA gene to amplify a specific region of the DNA was employed. The 16S rRNA contains both highly conserved and variable regions, which enables taxonomic classification [84].

In the study 2 by Ho *et al*. (2019) [25], slight decrease in α- diversity in the prebiotic group were found when compared to placebo. As mentioned earlier, α- diversity describes the microbial species diversity within the sample whereas, β-diversity describes the taxonomical. Prebiotics normally simulate growth of bacterias and an increase in bacterial diversity is expected after its intervention, but this was not found in this study. On the other hand, the measurement of β-diversity showed a significant effect indicating that prebiotic intervention impacted the overall structure of the gut microbiota changing relative abundances of different microbial taxa between prebiotic and placebo group.

Prebiotic intervention also showed an increase in bifidogenic effects, as indicated by a higher relative abundance of *Bifidobacterium spp*. at 3 months compared to the placebo group. The increase in *Bifidobacterium spp.* in prebiotic group may explain some of the improvements seen during the study. According to another study, children with T1D were associated with lower abundance in bacterias such as *Bifidobacterium* and *Lactobacillus.* The increase in abundance of beneficial bacterias, which produce SCFA, are beneficial for gut barrier integrity as they produce butyrate that is predominately used by colonocytes and can lead into an improvement in the tight junctions and decrease the overall IP [85].

A negative correlation was also observed between the relative abundance of *Terrisporobacter* and C-peptide. Despite limited knowledge about *Terrisporobacter,* it has been linked to oxidative stress and inflammation in humans. This suggests that a higher abundance of *Terrisporobacter* may be associated with lower insulin secretion in individuals with type 2 diabetes. Hence, we can assume that prebiotic supplementation may have a beneficial effect on insulin secretion by reducing the abundance of *Terrisporobacter* in the gut microbiota [25].

The bifidogenic effect found in the prebiotic group, during study 4 by Pedersen *et al*. (2016) [26] was close to significance(P = 0.0582). This result alongside the increase in richness found during the study provide insight as to how prebiotic supplementation may contribute with other beneficial properties than just a decrease in IP. No positive results were found from the study 6 by Ganda *et al*. (2020) [75]. This may be because dietary fibers are less capable of inducing an effect compared with other prebiotics such as inulin and GOS.

### Limitations

Overall, when comparing the 6 studies one with each other, various analytical techniques were utilized to measure the excretion of probes used such as L, M and ^51^Cr-EDTA, as some studies chose to use GS while others used LC–MS. The different techniques used may have difference in their sensitivity, calibration ranges and detection limits, which may lead to variation in the measured excretion percentages even if the IP remained constant. In future studies, it would be beneficial to have a standardized protocol using validated methods to establish some uniformity in conducted research, which would lead to more reliable comparisons across different studies.

In the studies that used L and M as the primary probes, a lack of defined limit to identify IP was found. While some studies such as [25] used values > 0.03 to define IP, others used value of L/M ratio > 0.08 as an indication on IP [1]. This lack of absolute reference value might be disadvantageous as the IP found during one study may not be defined the same in another study. Hence, in future research, it is important to determine a defined value to identify IP.

A lack of information regarding dietary interventions during the study was also observed, and this can have some impact on the results found by the studies. Prebiotics are also naturally found in various dietary sources such as onion, garlic and oats etc., which are widely consumed on daily basis. Without such information on dietary consumption, identifying the effects of actual prebiotic intake may be challenging across the studies, making it difficult to draw conclusions on whether differences in dietary consumption had any effect on the results obtained. Therefore, it is of importance that the researchers in future studies, collect and provide detailed information on dietary consumption.

The use of validated questionnaires were lacking in almost all of the studies. Questionnaires are highly beneficial while conducting clinical studies as they help determine the general state of health by measuring variabilities such as symptoms, pain, anxiety/depression of the individuals. IP is often associated with various symptoms such as abdominal discomfort and changes in the bowel movement. Observing any changes in these symptoms alongside potential psychological factors such as stress and anxiety can provide us with valuable insights into the potential non-measurable benefits of prebiotic usage. Therefore, for future studies on the effect of prebiotics on IP, it is important to develop/utilize validated questionaires.

## Conclusion

The interest in the connection between prebiotics and gut health is growing and investment in research involving the prebiotic-gut axis may provide pivotal guidance for the development of precision nutrition approaches to improve human health. This systematic review aimed to identify the potential effect of prebiotic intake on IP. Only one of the six studies reviewed showed a statistically significant improvement in IP (*p* > 0.05) after ingestion of prebiotics. Therefore, we conclude that limited support exists for the effect of prebiotics on IP improvement from current data and that more data is required to answer this question faithfully.

The current data also have several technical issues that may mask potential effects of prebiotics. Firstly, data sampling was too small, and most studies were underpowered for relevant statistical power, meaning studies were more likely to report false negatives. Secondly, the probes and/or measurement variables used to evaluate IP in studies did not always provide direct assessment of the gut barrier meaning conclusions were often drawn on indirect relationships. Using directly associated biomarkers such as GLP-2 and zonulin levels would enable clearer conclusions to be drawn about the potential improvements in IP without sole reliance on the urinary excretion of probes. It also provides a simpler method to measure the effect compared to artificial probes. Thirdly, there was a lack of standardized methods used for measurements such as sugar excretion. Adopting standardized protocols would establish more uniformity in the approach used for prebiotic clinical research, leading to easier cross-study comparisons and therefore more evidence-based conclusions. Likewise, defined values for the identification of IP were either lacking or often ill-defined amongst studies and participant questionnaires, meaning conclusions were drawn on limited data.

We recommend that future studies in prebiotics and IP use validated and standardized techniques to better investigate their relationship and potential beneficial outcomes for clearer conclusions to be drawn.

In regards of probes for measuring IP, the five most used carbohydrate derivatives (Table 1) vary considerably both in size, polarity, and in their solubility in water. More knowledge is needed on the correlation between the physiochemical properties of the molecules, their bioactive permeable conformation (the 3D total structure adopted in the crossing process), and hence their preferable characteristics and their actual permeability pathways. Clinical IP tests using multiple probes supported by conformational analysis for the various probes in their bioactive permeable conformation will provide more knowledge about the molecular complexity of intestinal IP and the pathway from the intestine to the urine via the blood. Finally, designing experiments with more defined prebiotics could unravel underlying IP mechanisms attribututed to individual types or groups of prebiotics that could then be harnessed for application of precision nutrition to improve human health. For example, breast milk feeding influenced IP in preterm infants [74]. Since breast milk contains HMOs and GAGs, further research on these particular prebiotics individually and in different combinations would provide results about the potential benefits for each class of these glycans.

## Supplementary Information

Below is the link to the electronic supplementary material.

Supplementary file1 (XLSX 44 KB)

## Data Availability

No datasets were generated or analysed during the current study.
